# AFM_1_ in Milk: Physical, Biological, and Prophylactic Methods to Mitigate Contamination

**DOI:** 10.3390/toxins7104330

**Published:** 2015-10-23

**Authors:** Laura Giovati, Walter Magliani, Tecla Ciociola, Claudia Santinoli, Stefania Conti, Luciano Polonelli

**Affiliations:** Department of Biomedical, Biotechnological, and Translational Sciences, Microbiology and Virology Unit, University of Parma, Parma 43125, Italy; E-Mails: laura.giovati@unipr.it (L.G.); walter.magliani@unipr.it (W.M.); tecla.ciociola@unipr.it (T.C.); claudia.santinoli@studenti.unipr.it (C.S.); stefania.conti@unipr.it (S.C.)

**Keywords:** aflatoxin, milk, biocontrol, enterosorption, biotransformation, vaccination

## Abstract

Aflatoxins (AFs) are toxic, carcinogenic, immunosuppressive secondary metabolites produced by some *Aspergillus* species which colonize crops, including many dietary staple foods and feed components. AFB_1_ is the prevalent and most toxic among AFs. In the liver, it is biotransformed into AFM_1_, which is then excreted into the milk of lactating mammals, including dairy animals. AFM_1_ has been shown to be cause of both acute and chronic toxicoses. The presence of AFM_1_ in milk and dairy products represents a worldwide concern since even small amounts of this metabolite may be of importance as long-term exposure is concerned. Contamination of milk may be mitigated either directly, decreasing the AFM_1_ content in contaminated milk, or indirectly, decreasing AFB_1_ contamination in the feed of dairy animals. Current strategies for AFM_1_ mitigation include good agricultural practices in pre-harvest and post-harvest management of feed crops (including storage) and physical or chemical decontamination of feed and milk. However, no single strategy offers a complete solution to the issue.

## 1. Introduction

Aflatoxins (AFs) are naturally occurring secondary metabolites produced mainly by toxigenic strains of *Aspergillus flavus* and *A. parasiticus* that colonize crops, including many dietary staple foods (maize, corn, groundnuts, and rice) and feed components. Contamination may occur either pre- or post-harvest and is more frequent in areas with a hot and humid climate [1]. However, contamination may also occur in temperate zones, when meteorological conditions combine with environmental factors and agricultural practices that favor the growth of toxigenic molds and AF production. Reports of AF contamination in the European continent are rising concurrently with the increase in the annual average temperature, reaching peaks during extreme weather conditions, as experienced during the summer season in 2003 and 2012 [2]. Within a group that includes more than 20 AFs and derivatives, B_1_, B_2_, G_1_, and G_2_ are the major naturally-occurring compounds. Among these, AFB_1_ is the most prevalent and toxic for man and animals [1]. AFs can enter humans or animals through ingestion, inhalation, or dermal contact [3,4,5,6], causing a wide range of adverse health effects collectively named as aflatoxicosis. According to AF dose and duration of exposure, acute or chronic aflatoxicoses may be recognized [7]. Major biochemical effects of AFs appear to be based primarily on their bioactivation to metabolites, which may interact with cellular organelles and macromolecules inducing the modification of normal metabolic and other vital processes. In particular, mutagenicity and carcinogenicity of AFB_1_ are associated with DNA binding properties of AFB_1_-8,9-epoxide, a highly electrophilic intermediate produced following biotransformation by the hepatic cytochrome P450 monooxygenases (CYP). AFB_1_ acute toxicity is also associated with protein binding properties of bioactive AFB_1_ metabolites, such as the AFB_1_ dialdehyde produced from the epoxide, which form adducts with protein amino groups, particularly lysine [8,9,10]. Binding to liver proteins may lead to organ failure, potentially resulting in acute aflatoxicosis. In addition, AFB_1_ mediated cytotoxicity and carcinogenicity may be due to oxidative damage induced in cells, tissues, and DNA [11].

In humans, major outbreaks of acute aflatoxicosis from contaminated food have been documented in developing countries, where AF contamination may be significant owing to meteorological conditions and deficiencies in detection, monitoring, and regulating measures to safeguard the food supply [7,12]. Hundreds of cases of acute aflatoxicosis, characterized by hemorrhage, acute liver damage, edema, and death, resulting from the consumption of extremely high doses of AFs have been described in India and Africa [13]. A more recent case-control study in Kenya demonstrated a clear association between AF levels in foods and risk of acute aflatoxicosis [14]. Although acute aflatoxicosis outbreaks sometimes result in a large number of fatalities, far more individuals and animals suffer from diseases associated with chronic exposure to low levels of AFs. It has been estimated that more than 4.5 billion people are chronically exposed to ingestion of AFs [7]. Following exposure assessments and molecular epidemiological studies, AFs have been classified as Group 1 carcinogens by International Agency for Research on Cancer [15]. In particular, based on ability to induce a specific mutation of *p53* gene [16], chronic consumption of AFs has been associated with the development of human hepatocellular carcinoma (HCC). Evidence also suggests a connection between chronic exposure to AFs and reduced uptake of nutrients from the diet, immunosuppression, and susceptibility to infectious agents such as *Plasmodium* spp. and HIV [17]. In children, chronic exposure to AFs is also associated with stunting, underweight, neurological impairment, immunosuppression, and mortality [7].

Collectively, these evidences illustrate the deleterious impact that chronic exposure even to low-level of AFs in the diet can pose for human health. Chronic exposition to AFs of livestock affects both animal health, increasing morbidity and mortality, and animal productivity, reducing growth, performance, reproductive capacity, response to vaccination, and production of eggs and milk [18]. Moreover, the biotransformation of ingested AFs could result in accumulation of their metabolites in different organs or tissues [19,20].

## 2. Carry-Over of AF in Milk

The term carry-over indicates the passage of undesired compounds from contaminated feed into food of animal origin. Evidence of carry-over due to AFs has been found in milk, porcine tissue, and eggs, representing an additional risk of human exposure to AFs, a potential cause of secondary aflatoxicosis [20]. In this perspective, the most threatening aspect of AF contamination of feed is related to carry-over of AFs in milk of dairy animals. The major AF metabolite excreted in milk in all species is M_1_ (AFM_1_). This product of mammalian bioconversion of part of the ingested AFB_1_ is formed by oxidative reactions catalyzed by hepatic CYP enzyme system, which lead to hydroxylation in the terminal furan ring of the parental molecule [21]. AFM_1_ represents the 95% of AFs detected in milk. Other metabolites, such as M_2_ (AFM_2_), aflatoxicol (AFL), M_4_ (AFM_4_), and Q_1_ (AFQ_1_), are detected in trace amounts and, thus, considered of less significance for public health [22,23]. AFM_1_ is normally detected in milk within 12 h of administration of AFB_1_-contaminated feed [24,25]. As a result of continuous daily exposure to constant levels of AFB_1_, the concentration of AFM_1_ in milk increases linearly for several days before finally achieving a steady-state, when an equilibrium between intake and excretion is established, and has been shown to decline as contaminated feed is withdrawn, reaching an undetectable level after 4–5 days [25,26,27,28,29]. The extent of carry-over in dairy cows is influenced by numerous nutritional and endogenous host factors, including breed, health of the animal, hepatic biotransformation capacity, lactation stage, and actual milk production [20]. Consequently, the excretion of AFM_1_ in milk may vary greatly between individual animals, from day to day, and from one milking to the next. From data obtained in different studies, the rate of AFB_1_ carry-over as AFM_1_ in milk of dairy cows was established to range from 0.3% to 6.2% [30]. Higher carry-over percentages are recorded in high-yielding cows, because of the significantly higher consumption of concentrated feeds [28,29,30,31,32,33,34].

## 3. Toxicity of AFM_1_

AFM_1_ is cause of both acute and chronic toxicoses, mainly through ingestion of contaminated milk and dairy products, or AFB_1_ metabolism in the liver [35]. Recent reports highlighted the occurrence in plants of AFM_1_, produced by *Aspergillus* spp. through a different biosynthetic pattern not involving AFB_1_, or possibly by insect pests metabolism from AFB_1_ [36,37,38] (Ezekiel *et al.*, 2012b; Streit *et al.*, 2013). In humans, exposure to AFM_1_ occurs mainly through consumption of milk [39]. Acute hepatotoxicity of AFM_1_ was initially observed in ducklings fed with AFM_1_ contaminated milk [40]. Subsequently, long-term studies in different animal species confirmed the hepatotoxicity of AFM_1_ and demonstrated its carcinogenic effect, although lower by about one order of magnitude as compared to AFB_1_ [39]. The relative carcinogenicity of AFM_1_ and AFB_1_ observed *in vivo* correlates with the metabolic activation to the respective epoxides observed in rat models and, *in vitro*, in murine and human liver microsomes [35,41,42]. The limited ability to metabolize AFM_1_ into the DNA-reactive epoxide may thus account for the reduced extent of DNA damage and pre-neoplastic lesions as compared to AFB_1_. AFM_1_ is mutagenic *in vitro* in *Salmonella typhimurium* strains [43] and shows the same *in vivo* genotoxic potential as AFB_1_ in *Drosophila melanogaster* [44], indicating a possible mutagenicity and genotoxicity also in mammalians *in vivo*.

Initially, AFM_1_ was categorized as group 2B human carcinogen by IARC [39]. Subsequent studies, improved in design, size, and accuracy of measurement of exposure biomarkers, allowed AFM_1_ reclassification as a group 1 human carcinogen [15]. While it was demonstrated that AFB_1_ must be converted into its reactive epoxide to bind protein and exert acute toxic effects [10], this process does not seem crucial to the cytotoxicity of AFM_1_. In human cell lines (MCL5), either expressing or not CYP enzymes, AFM_1_ demonstrated a direct toxic potential in absence of metabolic activation, in contrast to AFB_1_ [35]. More recently, AFM_1_ direct cytotoxicity was verified in cultured human intestinal enterocytes (Caco-2) [45,46]. Cytotoxic outcomes associated with intracellular reactive oxygen species (ROS) generation were also observed [45]. The above described findings suggest that exposure to AFM1 in milk may play an important causative role in observed cases of aflatoxicosis. The presence of AFM_1_ and its by-products in milk represents a worldwide concern as even small amounts of these metabolites may be of importance for consumers of large quantities of milk, like children, who are, moreover, more susceptible to the adverse effects of mycotoxins [47]. In Kenya, young children are weaned on to cow’s milk at an early age [48]; consumption of milk contaminated with AFM_1_ may reduce the development of their immune competence making them more susceptible to other diseases.

## 4. Regulation and Monitoring

AFs are considered as ubiquitous and unavoidable contaminants of foods and feeds [7]. Although it is difficult to remove AFs from human and animal diets, it is possible to decrease the risk of exposure through the establishment of regulatory limits and official monitoring plans to control the compliance of commodities with regulations through standardized analytical methods.

Considering the health risks associated with AFM_1_, many countries have established legal limits for maximum residue level (MRL) of AFM_1_ in milk [49]. These limits are not universal to all countries. The Commission of the European Community and the Codex Alimentarius Commission have set a MRL value of 50 ng/kg in raw milk. The MRL for AFM_1_ set by Southern Common Market (Mercosul) and US Food and Drug Administration is 500 ng/kg. In-between regulatory levels include the one from Syria, set at 200 ng/kg [49]. To avoid carry-over, MRL for AFB_1_ in feed of lactating cows have also been set, ranging from 5 μg AFB_1_/kg of feed (European Community) to 10 μg/kg (China) and 20 μg/kg (USA) [49]. The rationale for the establishment of specific regulations in each country varies widely, depending on factors such as results of risk analysis (toxicological data and information on susceptible commodities), analytical capabilities (sampling and detection limits), and socio-political issues (adequacy of food supply, economic condition of a country, trade requirements) [50]. Developing countries have poor food safety and animal health systems not allowing compliance with strict regulatory limits; legislation is often applied only to export commodities [51]. Products not matching with export requirements are likely sold for local consumption; therefore, the testing of exported commodities will actually undermine the food safety for the local market. In developing countries of Asia and Africa, lenient standard limits for AFM_1_ (and AFs in general) and economic constraints for monitoring programs have been connected with the high prevalence rate of liver cancer [52].

Efforts should be made in attempting to gain further and extensive knowledge on human health hazards related to long term exposure to low-level AFM_1_, providing scientific basis to standardize the already-existing regulatory limits and to implement policies to reduce contamination in low-resource countries.

Nevertheless, implementation of regulatory and monitoring measures do not represent a definitive solution in control of AFM_1_ contamination of milk because of multiple hurdles. For example, regulatory limits for MRL prevent exposure to high contaminations, without removing AF from the food chain, so that low-level exposure or synergistic interactions with other mycotoxins cannot be excluded [53]. Another hurdle is that products exceeding MRL are subject to seizure. This implies costs in terms of lost revenue and additional costs for properly disposing the contaminated commodity. Finally, the MRL definition, as well as compliance with regulation, is restricted by limits of detection, analytical accuracy and sampling difficulties that may be due to heterogeneity of AF distribution in different lots.

## 5. Mitigation of AFM_1_ Occurrence in Milk

To minimize risks associated with unavoidable exposure to AFs, regulation and monitoring measures must be supported by in field (pre-harvest) and storage (post-harvest) interventions which may be applied to minimize AF contamination. AFM_1_ is excreted in milk of dairy animals following metabolism of AFB_1_ ingested with feed. Contamination of milk may, thus, be reduced either directly, decreasing AFM_1_ content of contaminated milk, or indirectly, decreasing AFB_1_ contamination in feed of dairy animals. Many methodologies developed to reduce AFM_1_ contamination with both direct and indirect approaches have been extensively reviewed [54]. These include good agricultural practices in pre-harvest and post-harvest management of feed crops (including storage) and physical or chemical decontamination/detoxification of feed and milk. Beyond ongoing research to improve efficiency, safety, and reliability of these interventions, there is a growing interest in developing environmental friendly, cost-effective, and specific alternatives for AFM_1_ mitigation. Highly promising technologies proposed for this scope exploit microorganisms, purified microbial enzymes, dietary clay minerals, and specific antibodies induced by vaccination. In the following paragraphs, we describe some applications of these technologies to reduce the concentration of AFB_1_ in feed, the bioavailability of AFB_1_ or its metabolites in the body, or the carry-over as AFM_1_ in milk.

### 5.1. Biological Control

Biological control (biocontrol) may be implemented to reduce AFB_1_ concentrations in feed of dairy animal during both crop development and post-harvest storage, indirectly reducing AFM_1_ contamination of milk.

#### 5.1.1. Biocontrol in Field

A biocontrol agent may physically destroy a pest, secrete a toxin that destroys the pest, or out-compete the pest in its ecological niche. In the field, biocontrol of AFs refers to the use of organisms to reduce the incidence of toxigenic strains of *Aspergillus* in susceptible crops, thus reducing AF contamination. Different organisms, including bacteria, yeasts, and nontoxigenic *Aspergillus* strains, have been tested as competitive biocontrol agents. Bacteria isolated from soil have shown a good potential as biocontrol agents under laboratory conditions. In one experiment, *Rhodococcus erythropolis* completely inhibited mycelial growth and AFB_1_ production by *A. flavus*, while *Bacillus subtilis* and *Pseudomonas fluorescens* reduced mycelium growth in a range of 68% to 93% and AFB_1_ production from 58% to 83.7% [55]. A number of microorganisms isolated from maize fields (*B. subtilis*, *Lactobacillus* spp., *Pseudomonas* spp., *Ralstonia* spp., and *Burkholderia* spp.) effectively inhibited *A. flavus* growth *in vitro*; *B*. *subtilis* and *P*. *solanacearum* were able to inhibit both fungal growth and AF accumulation. However, they did not show good efficacies in field conditions, mainly because of difficulties in the application of the bacterial cells to the *Aspergillus* infection sites [56,57]. Similarly, saprophytic yeasts (*Candida krusei* and *Wickerhamomyces anomalus*) inhibited AF production by *A. flavus in vitro*, but further research is needed to assay their potential for AF reduction under crop production conditions [58].

To date, the most successful biocontrol method employs nontoxigenic strains of *A. flavus* and *A. parasiticus*, applied with a carrier/substrate, such as a small grain, in fields where they competitively exclude the toxigenic strains and preferentially infect the susceptible crop [59]. Non-aflatoxigenic native *A. flavus* has been effective in significantly reducing AF contamination in fields of maize, groundnuts, and cottonseed [60,61,62,63,64]. The biocompetitive *A. flavus* strain NRRL21882, which has been developed for controlling AF contamination in peanuts, was registered in 2004 as a biopesticide by the US Environmental Protection Agency with the name of Afla-Guard^®^. During the first year of application in selected fields, Afla-Guard^®^ changed the composition of *A. flavus* soil populations from an average of 71.1% of toxigenic strains in untreated fields to 4.0% in treated soils. A consistent reduction in AF contamination in peanuts from fields treated with Afla-Guard^®^ was also observed [65]. This technology is now utilized in cotton and maize fields in USA and Kenya, and several reports indicate that *A. flavus* is able to reduce AF levels in treated *versus* untreated fields as much as 20-fold [66]. Importantly, other than in USA and Kenya, native atoxigenic *A. flavus* strains have been shown to effectively reduce AF production in maize and peanuts in Africa, Australia, and China, indicating that nontoxigenic strains for the control of AF contamination could be applied in different agro-ecozones [63,67,68,69]. The application of atoxigenic strains in field may also offer a post-harvest advantage by lowering concentration of toxigenic strains carried with crops. During storage, the atoxigenic strains dwelling in the crop may continue to offer protection. In fact, some evidence suggests that pre-harvest introduction of biocontrol *A. flavus* is able to reduce levels of AFs even in poorly-stored maize [70].

#### 5.1.2. Biocontrol during Storage of Feed

Post-harvest biological control of AFs exploits the antagonistic ability of probiotic microorganisms inoculated in stored commodities to impair growth and AF production of phytopathogenic fungi, or to reduce AF content through binding [71]. Biocontrol to counteract AF contamination during storage has been tested with some success with probiotic yeast and bacterial strains. *Saccharomyces cerevisiae* resulted to be one of the most effective microorganisms for binding AFB_1_ [72]. *S. cerevisiae* YEF 186 was tested as an antagonist of *A. parasiticus* in two peanut cultivars (IAC Runner 886 and IAC Caiapó) [73]. The inoculation of *S. cerevisiae* 3 h before the pathogen was shown to decrease AFB_1_ concentration by 74.4% and 55.9% after seven and 15 days of incubation, respectively. This reduction was probably due to AF adhesion to the yeast cell wall or to AF degradation by the yeast. *S. cerevisiae* RC008 and RC016 proved effective at reducing *in vitro* growth of *A. parasiticus* and AFB_1_ production in different environmental conditions, related to that found in stored feedstuff [74].

Lactic acid bacteria (LAB) belonging to the genera *Lactococcus* and *Lactobacillus* have been also investigated for their ability as biocontrol agents *versus* aflatoxigenic *A. flavus* [75]. *Lactobacillus plantarum*, *L. fermentum* and *L. delbrueckii* significantly inhibited growth and AFB_1_ production of *A. flavus* [76]. Similarly, two *Lactobacillus* strains (*L. rhamnosus* L60 and *L. fermentum* L23) with known probiotic activities were both able to inhibit the mycelia growth of ten co-cultured aflatoxigenic *Aspergillus* strains and to reduce AFB_1_ production (95.7%–99.8% reduction with L60 and 27.5%–100% with L23) [77]. Reduction of AF production may relate to inhibitory low-molecular-weight metabolites produced by LAB at the beginning of the exponential phase of growth [75]. *L. rhamnosus* GG and *L. rhamnosus* LC705 were shown to eliminate AFB_1_ from the culture medium by physical adsorption [78]. Probiotics are “Generally Recognized as Safe” (GRAS) microorganisms, which are allowed in food without any restriction. The above-described experiments suggest that probiotics could be used in the food and feed industries for the biological control of AF production or dispersion by toxigenic *Aspergillus* strains, conferring protection during storage and providing an additional positive effect in the digestive tract of consumers. LAB (*L. plantarum* and *L. fermentum*) are widely used as microbial inoculants in silage production as economical and practical alternatives to acid-based additives, for improving or guaranteeing aerobic stability [79,80]. Novel products for animal feed could include LAB inoculants with both probiotic and biocontrol properties, able to improve stability of silage, prolong the safe storage of feedstuff, and exert beneficial properties after animal consumption.

Reduction of AF and spore production by *A. parasiticus* IMI 242695 in the presence of yeast and bacterial agents (*S. cerevisiae* var. *boulardii*, *S. cerevisiae* UFMG 905, and *L. delbrueckii* UFV H2b20) was evaluated [81]. When inoculated in pairs, all probiotic combinations significantly reduced AF production, remaining viable in high numbers for a prolonged time, comparable to typical storage period of commodities [81]. The possible impact of LAB and yeasts, added to reduce AFs, on the organoleptic characteristics of the products and the exact mechanisms of reduction of AF content need to be further clarified.

### 5.2. Enterosorption by Dietary Clay Minerals

Adsorbing agents are compounds that can be included in food or feed, or taken separately during mealtimes, to reduce AF absorption in the gastrointestinal tract, consequently reducing further steps of toxin distribution and metabolism in organs and tissues [24,82]. Several clay materials, including activated charcoal, bentonite, zeolite, and hydrated sodium calcium aluminosilicate (HSCAS), present varying abilities to bind AFs *in vitro*. However, the binding is not often effective in preventing uptake from the digestive system *in vivo* and the efficiency in preventing aflatoxicosis varies with the adsorbent [83]. Selected HSCAS have proven to be the most highly selective and effective enterosorbents. An HSCAS (marketed as NovaSil^®^), initially sold as an anticaking additive for animal feeds, presented effective AF binding abilities both *in vitro* and *in vivo*, showing a positive correlation between efficacies in the two models. HSCAS showed adsorption of AFB_1_ with high affinity and reduction of its bioavailability in poultry. In subsequent studies, HSCAS and other similar montmorillonite and smectite clays have shown to prevent aflatoxicosis in multiple animal models, when included in the diet, by binding AFs with high affinity and high capacity in the gastrointestinal tract [84].

#### 5.2.1. Clay-Based Decontamination of Feed

Inclusion of enterosorbents in the diet of dairy animals may reduce absorption of AFB_1_ in the animal body, preventing further steps of toxin distribution and metabolism, thus reducing carry-over in milk. Significant reductions of the concentration of AFM_1_ in milk were observed when clay enterosorbents were included in the diet of lactating dairy cattle and goats fed with feed contaminated with AFB_1_ [84]. In dairy cows, activated carbon (AC) and HSCAS, mixed to AFB_1_ contaminated feed with an inclusion rate of 2%, reduced AFB_1_ carry-over as AFM_1_ in milk of 50% and 36%, respectively [31]. HSACS at 1% resulted in a carry-over reduction of 24% [34]. A study comparing the effects of AC, esterified glucomannan, calcium bentonite, and three HSCAS products showed reductions in milk AFM_1_ concentrations of 5.4%, 59%, 31%, 65%, 50%, and 61%, respectively [24]. The inclusion of two commercial HSCAS products, Novasil Plus^®^ and Solis^®^, or an esterified glucomannan product (MTB-100) at 0.5% to the diet of dairy cows reduced milk AFM_1_ concentration by 45%, 48%, and 4%, respectively [85]. More recently, a 17% reduction in milk AFM_1_ was observed in response to HSCAS at 1% [86]. The addition of bentonite (AB-20) to the diet of cattle reduced by 60.4% AFM_1_ concentration in the milk of cows fed an AF-contaminated diet [87]. Although there may be some possible risks or adverse effects to be considered, such as possible reduction of bioavailability of some nutrients in feed, different adsorbent clays are commercially available and bentonite was approved by the European Commision as a feed additive for the reduction of the contamination by AFB_1_ [88]. The use of clay-based enterosorbents as feed additives is one of the most prominent approaches to reduce the risk for aflatoxicosis in dairy animals, and to minimize AFB_1_ carry-over as AFM_1_ from contaminated feeds into milk.

#### 5.2.2. Clay-Based Decontamination of Milk

Clay enterosorbents have also been proposed as for direct decontamination of AFM_1_ in milk, although, with regard to this approach, the line between AF mitigation and classic food adulteration is very fine. Early experiments demonstrated good reductions with bentonite [89]. More recently, the ability of saponite-rich bentonite to reduce AFM_1_ contamination in milk was investigated. The detoxification capacity of the bentonites used was efficient, bringing contamination below the European standard limits for AFM_1_ (50 ng/kg), with moderate alteration of the nutritional properties of the milk. Bentonite residues retained in milk (0.4%) were of no concern for human health [90]. Information on other parameters of milk quality is scarce. Further investigations on the possibility to separate adsorbent-bound toxin from milk are in progress.

### 5.3. Microbial Enterosorption

Microbial enterosorption exploits yeast and bacteria as biological adsorbents. Probiotics, such as LAB and *Saccharomyces* spp., are the most frequently employed binding agents, due to their GRAS status, high binding abilities, and wide distribution in nature. Beyond the ability to prevent aflatoxigenic mold growth and synthesis of AFs in stored food, as previously discussed, LAB demonstrated a significant potential to remove AFB_1_ from liquid media with strain- and dose-dependent efficiency [91]. Killed bacteria showed an enhanced ability to remove AFB_1_ suggesting that, since metabolic activation is not necessary, binding may explain the interaction between AFB_1_ and LAB [92].

Five *Lactobacillus* strains (*L. rhamnosus* GG, *L. rhamnosus* LC705, *L. acidophilus*, *L. gasseri*, and *L. casei*) bound AFs *in vitro.* In particular, the probiotic strains *L. rhamnosus* GG and *L. rhamnosus* LC705 were very effective in immediately removing as much as 80% (*w*/*w*) of AFB_1_ [78]. Reversibility of binding suggested non-covalent interactions between AFB_1_ and hydrophobic pockets on the bacterial surface [78]. Further studies proved that other LAB strains were able to bind and remove AFB_1_ in liquid media in a concentration-dependent manner [93,94].

More recently, it has been proposed to exploit microbial enterosorption to reduce residual levels of AFM_1_ in contaminated milk. Commercial *Lactobacillus* and *Streptococcus* strains were able to reduce to varying degrees AFM_1_ concentration in phosphate buffered saline (PBS), milk, and yoghurt [95,96,97]. In particular, 28% and 39% of AFM_1_ in milk samples were bound by *L. bulgaricus* CH-2 and *S. thermophilus* ST-36, respectively, while lower levels of AFM_1_ binding were reported in yoghurt [97]. The ability of different LAB to remove AFM_1_ from processed milk, such as yoghurt, was also demonstrated [98]. The binding of AFM_1_ by microbial cells has been reported as a rapid process [99,100]. The variable binding efficiencies of AFM_1_ by microbial cells are thought to be due to differences in the structure of cell walls and membranes, incubation time and temperature, AFM_1_ levels, and pH [95,101]. Maximum AFM_1_ binding capability (100%) has been reported with a combination of *S. cerevisiae* and a pool of three heat-killed LAB [102]. Interestingly, the use of killed bacteria may be an advantage, as viable microorganisms may result in spoilage of milk and milk products by undesired fermentation. Further investigations are required to determine the stability of the AFM_1_-microbial cell complexes and the amount of AFM_1_ available for intestinal absorption (bioaccessibility) before commercial application in the dairy industry. However, microbial enterosorption of AFM_1_ is a promising strategy to reduce or eliminate chronic low-level exposure to AFM_1_ in milk by effective and specific natural binders which may also deliver positive health effects as probiotics.

### 5.4. Biotransformation by Microorganisms or Enzymes

Biotransformation, or biodetoxification, utilizes microorganisms and/or their purified enzymatic products to catabolize the AF molecule, or to transform or cleave it to less or non-toxic compounds. Biotransforming agents used as additives in contaminated feed could detoxify AFB_1_, decreasing its carry-over in milk as AFM_1_. To be used as animal feed additives, biotransforming agents must (i) rapidly degrade AFs into non-toxic metabolites, under variable oxygen conditions, and in a complex environment, and (ii) prove safe for animals [54].

Many reports show the degradation of AFs by fungi and bacteria with varied efficiencies. Several fungal strains, including a non-toxigenic *A. flavus*, *A. niger*, *Eurotium herbariorum*, and a *Rhizopus* sp., have been found to biotransform AFB_1_ into less toxic metabolites [103]. However, their potential use in the food industry may be limited by the long incubation time required for detoxification (more than 72 h), incomplete degradation, non-adaptation to typical food fermentations, and culture pigmentation [104]. Some of these strains may also produce AFB_1_ under varying conditions [105]. Bacteria investigated for their AF biotransforming abilities include soil bacteria like *Nocardia corynebacterioides* [106], *Mycobacterium* *fluoranthenivorans* [107], *Rhodococcus erythropolis* [108], *Stenotrophomonas maltophilia* [109], *Myxococcus fulvus* [110], among others. Interesting results have been obtained with *N. corynebacterioides*, tested on contaminated milk and several solid substrates, including feed, which showed a complete detoxification of AFs (AFB, AFG, and AFM), without production of new toxic molecules [106]. Further studies with ^14^C-AFB_1_ revealed that the radioactive molecules were partially metabolized and partially adsorbed to *N. corynebacterioides* cells, while cation chelators influenced the detoxification capacity of *N. corynebacterioides*, suggesting an enzymatic involvement in the degradation process [111]. *M. fluoranthenivorans* was found to be capable of degrading AFB_1_ as a single carbon source, detoxifying 70%–80% of the initial concentration within 36 h and 100% in 72 h [107]. Cell extracts of *M. fluoranthenivorans* degraded about 90% of the initial amount of AFB_1_ in 4 h, while no AFB_1_ was detected after 8 h [112]. Rapid and effective degradation of AFB_1_ was also shown by *R. erythropolis* (83% in 48 h and 97% in 72 h) and extracellular extracts from *R. erythropolis* (68% AFB_1_ reduction in 72 h) [108]. Efficient AFB_1_ degradation at different temperatures was also observed in both *R. erythropolis* and *M. fluoranthenivorans*. High degradation rates and wide temperature ranges for AFB_1_ degradation indicate a potential and promising application for AFB_1_ detoxification in the food and feed industry.

Some studies further explored the role of enzymes in AFB_1_ biotransformation [113,114]. AF detoxification enzymes such as laccase, lactoperoxidase, and anti-oxidative stress enzymes have been identified from bacteria (*M. fulvus*) [115] and edible fungi [71,116]. In one study, the screening of extracellular enzymes in white-rot and brown-rot fungi led to the purification of a protein from *Pleurotus ostreatus* with good AF-degradation activity [114]. Since *P. ostreatus* is a non-toxic, edible fungus, the purified enzyme is a promising candidate for the degradation of AFs in foods and feeds. AFB_1_ biotransformation enzymes, once characterized, could be mass-produced and used for the treatment of materials contaminated with AFB_1_.

### 5.5. Neutralization by Specific Antibodies Induced by Vaccination

AFs in diets of cattle have been reported to increase morbidity and mortality, and to reduce milk yield and quality [117]. Vaccination of dairy animals against AFB1 could supply a solution to address the issue of AF contamination in both nutrition safety and animal health perspectives, increasing milk safety and reducing negative effects on animal health and productivity of chronic exposition of livestock to AFs. An entirely innovative strategy to decrease risks associated with contamination of feeds by AFs, and their carry-over in milk and edible tissues, could rely on vaccination to induce antibodies (Abs) that specifically block initial absorption or bioactivation of AFs, toxicity, and/or excretion in milk or other products, by immuno-interception (neutralization). Systemic vaccination of dairy cows and heifers has recently proved to be effective in reducing AFB_1_ carry-over as AFM_1_ in milk [118,119]. In these experiments, cattle were vaccinated systemically with a mycotoxoid vaccine formulated with protein-conjugates of Anaflatoxin B1 (An-AFB_1_), a chemically detoxified form of AFB_1_, showing the ability to induce Abs reactive with the parent molecule. In a first experiment, vaccination of cows elicited a persistent titer of anti-AFB_1_ Abs, which also cross-reacted with AFG_1_, AFB_2_, and AFG_2_. Following the evaluation of anti-AFB_1_ Ab titer, 50% of the vaccinated cows could be categorized as “high responder” animals. The cows were exposed to continuous feeding with an AFB_1_-contaminated diet in two trials, performed in different stages of the milk production cycle (mid- and late-lactation stage). In both trials, AFM_1_ concentrations in milk of vaccinated cows were significantly lower than in milk of unvaccinated controls and the efficacy of specific Abs in reducing carry-over into milk was proportional to their titer. In particular, high responder cows produced an average milk AFM_1_ concentration 46% and 37% lower compared to that observed in control cows during mid- and late-lactation stage, respectively [118]. Importantly, reductions of AFM_1_ concentration obtained in different stages of lactation suggest that vaccination may confer a protection over the whole production cycle, before the drying off period. Subsequent experiments analyzed the effect of conjugation of An-AFB_1_ with other protein carriers, the effect of administration with various immunological adjuvants, and the effect of animal age on specific anti-AFB_1_ Ab titers [119]. The results suggested that pre-calving administration could increase the effectiveness of vaccination, resulting in 100% high responder cows. Anti-AFB_1_ Ab titers in vaccinated heifers decreased during pregnancy and after calving, and returned to previous levels after one booster dose at the beginning of the milk production cycle. Monitoring of AFM_1_ concentrations in milk of vaccinated and control cows, demonstrated the effectiveness of anti-AFB_1_ Abs in reducing from 3.40% to 0.78% the carry-over of AFB_1_ as AFM_1_, resulting in a 74% reduction of AFM_1_ contamination.

Overall, these results indicate that vaccination may represent a valuable tool for prevention of AF carry-over, contributing significantly to the safety of milk and dairy products. Evidence also exists that vaccination may reduce health hazard of AF contamination of feed for livestock. Successful attempts of immunizing rodents and chickens to induce a humoral response specific to AFB_1_ have been described [120,121,122,123]. Lower mortality and reduction of acute toxic effects in the liver were also demonstrated in immunized rabbits and rats challenged with a single dose of AFB_1_ [120,121,122,123]. Following similar immunization experiments, a reduction of the covalent binding of AFB_1_ to liver DNA was observed, indicating a possible reduced susceptibility for liver tumor formation [124]. Additional *in vitro* investigations proved that specific anti-AFB_1_ antisera were able to decrease genotoxic and mutagenic effects of AFB_1_ [125,126]. Although some other factors, such as the fate of AFB_1_ captured by Abs, should be further investigated, the results of these studies suggest that vaccination against AFB_1_ could be used to protect animals against aflatoxicosis.

## 6. Conclusions

Despite international efforts, prevention and control of AF contamination of food and feed remains difficult. Good agricultural practices during both pre- and post-harvest, as well as monitoring activity towards AF contamination of feed, minimize but not eliminate the risks of contamination of milk with AFM_1_. In fact, numerous studies report a widespread AFM_1_ contamination in milk and milk products, particularly in the developing countries [127]. Due to the wide health and economic implications associated with milk safety and chronic exposure to AFM_1_, different lines of investigation are being pursued and extended to develop innovative and more effective intervention strategies to mitigate AFM_1_ risks. We described some interventions that exploit microorganisms, purified microbial enzymes, dietary clay minerals, and specific Abs induced by vaccination to reduce directly or indirectly AFM_1_ contamination of milk. Proposed interventions could be delivered at agricultural (in the field or post-harvest), dietary (feed processing or supplementation), or immunoprophylactic levels (vaccination) acting at different critical points along the milk and milk-derived food production chain. Each of the presented methods has benefits and drawbacks and no one emerges as a definitive, standalone solution to prevent AFB_1_ carry-over as AFM_1_ from feed to milk. Rather, they appear as a pool of interventions that could be implemented as a part of a potential comprehensive prevention and control plan for food safety and quality assurance to reduce health impacts and trade losses connected to AFM_1_ contamination of milk.
